# Profiling Receptor Tyrosine Kinase Fusions in Chinese Breast Cancers

**DOI:** 10.3389/fonc.2021.741142

**Published:** 2021-09-28

**Authors:** Zhonghua Tao, Jianxia Liu, Ting Li, Hong Xu, Kai Chen, Jian Zhang, Hao Zhou, Jie Sun, Jinming Han, Zhaoji Guo, Hua Yang, Wen-Ming Cao, Xichun Hu

**Affiliations:** ^1^ Department of Medical Oncology, Fudan University Shanghai Cancer Center, Shanghai, China; ^2^ Department of Oncology, Shanghai Medical College, Fudan University, Shanghai, China; ^3^ Department of General Surgery, The First Affiliated Hospital of Soochow University, Suzhou, China; ^4^ Department of Oncology, The First Affiliated Hospital of Soochow University, Suzhou, China; ^5^ Department of Oncology, The First Affiliated Hospital of Zhengzhou University, Zhengzhou, China; ^6^ Department of Medical Oncology, Affiliated Hospital of Hebei University, Baoding, China; ^7^ Department of Breast Medical Oncology, Cancer Hospital of the University of Chinese Academy of Sciences (Zhejiang Cancer Hospital), Hangzhou, China

**Keywords:** receptor tyrosine kinase, gene fusion, genomic rearrangments, breast cancer, next-generation sequencing

## Abstract

**Background:**

Receptor tyrosine kinases (RTKs) are a class of tyrosine kinases that regulate cell-to-cell communication and control a variety of complex biological functions. Dysregulation of RTK signaling partly due to chromosomal rearrangements leads to novel tyrosine kinase fusion oncoproteins that are possibly driver alterations to cancers. Targeting some RTK fusions with specific tyrosine kinases inhibitors (TKIs) is an effective therapeutic strategy across a spectrum of RTK fusion-related cancers. However, there is still a paucity of extensive RTK fusion investigations in breast cancer. This study aims to characterize RTK fusions in Chinese breast cancer patients.

**Methods:**

An in-house DNA sequencing database of 1440 Chinese breast cancer patients with a capture-based panel (520 gene or 108 gene-panel) was thoroughly reviewed. A total of 2,229 samples including 1,045 tissues and 1,184 plasmas were analyzed. RTK fusion was defined as an in-frame fusion with the tyrosine kinase domain of the RTK completely retained. Concomitant mutations were also analyzed and tumor mutational burden (TMB) was calculated. Patients’ clinical characteristics were retrieved from case records.

**Results:**

A total of 30 RTK fusion events were identified from 27 breast cancer patients with a prevalence of 1.875%%. *FGFR2* fusions were seen the most commonly (n=7), followed by *RET* (n=5), *ROS1* (n=3), *NTRK3* (n=3), *BRAF* (n=2), and *NTRK1* (n=2). Other *RTK* fusions including *ALK, EGFR, FGFR1, FGFR3*, *MET*, and *NTRK2* were identified in one patient each. A total of 27 unique resultant fusion proteins (22 with a novel partner) were discovered including 19 intrachromosomal rearrangements and 8 interchromosomal ones. Twenty-one fusions had the tyrosine kinase domain in-frame fused with a partner gene and six were juxtaposed with an intergenic space. Among the 27 fusions, *FGFR2-WDR11* (E17: intergenic) (n=3) and *ETV6-NTRK3* (E5:E15) (n=2) occurred recurrently. Of note, the normalized abundance of RTK fusion (fusion AF/max AF) correlated negatively with TMB (r=-0.48, P=0.017). Patients with TMB < 8 (Mutations/Mb) displayed a higher fusion abundance than those with TMB ≥ 8 (Mutations/Mb) (P=0.025). Moreover, *CREBBP* mutation only co-occurred with *FGFR2* fusion (P=0.012), while *NTRK3* fusion and *TP53* mutation were mutually exclusive (P=0.019).

**Conclusion:**

This is the first study comprehensively delineating the prevalence and spectrum of RTK fusions in Chinese breast cancers. Further study is ongoing to identify the enriched subpopulation who may benefit from RTK fusion inhibitors.

## Introduction

Receptor tyrosine kinases (RTKs) are a subclass of tyrosine kinases that share a similar protein structure comprised of an extracellular ligand-binding domain, a transmembrane helix, and a tyrosine kinase domain (TKD)-included intracellular region (1). Approximately 58 RTK genes grouped into 20 subfamilies have been found in the human genome (2). RTKs regulate cell-to-cell communication and control a variety of complex biological functions, such as cell growth, differentiation, and metabolism (3). The RTK activity is tightly regulated under normal physiologic conditions. Dysregulation of RTK signaling leads to a number of human diseases, most notably, cancers. Constitutive activation of RTK can be caused by gain-of-function mutations, genomic amplification, or chromosomal rearrangements (4). It may confer oncogenic properties on normal cells therefore trigger RTK-induced tumorigenesis.

As one of the mechanisms mediating abnormal RTK activation in cancers, chromosomal rearrangements can result in the formation of novel tyrosine kinase fusion oncoproteins that are often therapeutically targetable with small molecule inhibitors. It has been proven that inhibiting RTK fusions with specific tyrosine kinases inhibitors (TKIs) is an effective therapeutic strategy across a spectrum of RTK fusion-driven cancers. These targetable RTK rearrangements consist of *ALK* fusion in lung cancer (5) and anaplastic large cell lymphoma (6), *ROS1* fusion in lung cancer (7) and glioblastoma (8), *RET* fusion in lung (9) and thyroid cancer (10), *FGFR* fusion in bile duct (11) and urothelial carcinoma (12), as well as *NTRK* fusion in pan-cancer (13).

As a highly heterogeneous disease, breast cancer comprises distinct molecular subtypes with varied clinical outcomes (14). Patients with advanced breast cancers that are negative for both estrogen-receptor and human epidermal growth factor receptor 2 (ER-/HER2-) have very limited therapeutic options. On the other hand, although endocrine therapy and HER2-targeted therapy have achieved great success in treating ER+ or HER2+ breast cancers, approximately 50% of the advanced cases develop resistance to these treatments (15–17). Therefore, exploring RTK fusions in breast cancer may drive the discovery of novel therapy that will bring these refractory patients more treatment opportunities. Despite the research in RTK fusions has driven the approval of relevant targeted therapies in a variety of cancer types, similar investigations remain limited in breast cancer. *ETV6-NTRK3* fusion, initially described in congenital fibrosarcoma and mesoblastic nephroma, was identified as a primary oncogenic event in human secretory breast carcinoma by RT-PCR and fluorescence *in situ* hybridization (FISH) decades ago (18, 19). Wu et al. focused on *FGFR* gene fusions in diverse cancers and identified *FGFR2-AFF3*, *FGFR2-CASP7*, *FGFR2-CCDC6* and *ERLIN2-FGFR1* in breast cancer (20). Paratala and colleagues profiled *RET* fusions in breast cancer and identified *CCDC6-RET*, *NCOA4-RET* and *RASGEF1A-RET* (21). Of note, there is still a paucity of data comprehensively characterizing RTK gene fusions in this disease so far, especially in the Eastern Asian population.

In this study, we aim to delineate potentially targetable RTK fusions in Chinese breast cancer patients and to explore their associations with clinical and other genetic characteristics.

## Materials and Methods

### Patients’ Information and Study Design

An in-house DNA-based next-generation sequencing (NGS) database of 1440 Chinese breast cancer patients was retrospectively reviewed for RTK gene fusions. The median age of the 1440 patients was 50 years. 11% of them had metastatic disease, 32.2% were at early stage, and 56.8% had clinical stage unavailable. All recruited patients had their tissue or plasma samples somatic mutation profiled for genetic testing and treatment selection from 2016 to 2020 by capture-based sequencing using a 520 gene-panel (n=1,014) or a 108 gene-panel (n=426) in a Clinical Laboratory Improvement Amendments (CLIA)/CAP-certified laboratory (Burning Rock Biotech, Guangzhou, China). A total of 684 patients had multiple samples sequenced and 2,229 samples including 1,045 tissues and 1,184 plasmas were analyzed.The sequencing depth was >1000X for tissues and >10000X for plasmas. The 20% mean depth coverage was >95%. RTK genes analyzed in this study included *ALK, BRAF, EGFR, FGFR1, FGFR2, FGFR3, MET, NTRK1, NTRK2, NTRK3, RET and ROS1*, which are commonly involved in cancer genome rearrangements. RTK fusions with potential functionality were identified, defined as an in-frame fusion with the intact tyrosine kinase domain of the RTK gene retained. Concomitant genomic alterations and tumor mutational burden (TMB) were also analyzed and calculated if applicable. Patients’ clinical characteristics were retrieved from case records. The RTK fusion prevalence was also compared with MSKCC (22) and TCGA (23) databases. The study was approved by the institutional review board (IRB) of Fudan University Shanghai Cancer Center. All patients had completed written informed consents before they received the genetic testing, giving the permission to use their archived samples and relative information for scientific research in the further. Due to the retrospective nature of the study, the requirement for informed consent for this study was exempted by the IRB.

### NGS Data Analyses

Sequencing data were analyzed as previously described (3). Briefly, by using the BWA aligner 0.7, data in FASTQ format were aligned to the reference human genome (hg19). Local alignment optimization, duplication marking, and variant calling were conducted using the Genome Analysis Tool Kit v.3.2 (24) and VarScan v.2.4.3 (25). Low quality variants with depth <50× or mutated allele reads <8× were filtered out. Variants with a frequency >0.1% in the databases (ExAC, 1,000 Genomes, dbSNP, or ESP6500SI-V2) were excluded from further analysis. The remaining variants were annotated with ANNOVAR (2016-02-01 release) (26) and SnpEff v.3.6 (27). Stuctural variation was analyzed using an in-house script markSV (CN112349346A). The algorithm was based on two structure variation signalings: soft clipped reads and paired end reads. The copy number variation (CNV) was estimated with an in-house algorithm based on the sequencing depth as described previously (28).TMB per patient was calculated as the ratio between the total number of nonsynonymous mutations detected with the coding region size of the panel. The relative RTK fusion allele frequency (RTK. RAF) was calculated as the ratio of fusion allele frequency by the maximum allele frequency of a given sample (fusionAF/max AF).

### Statistical Analyses

Statistical analyses were performed using R version 3.3.3 software. Differences in the groups were calculated and presented using Fisher’s exact test, paired two-tailed Student’s t-test, or analysis of variance as appropriate. Pearson correlation was performed to study the correlation between TMB and the RTK. RAF. P-values <0.05 were considered statistically significant.

## Results

### Patients’ Characteristics

A total of 27 patients with breast cancer were identified with putatively functional RTK fusions. The median age of this RTK fusion-positive cohort was 52 years (**Table 1**). Triple-negative breast cancer subtype (TNBC) comprised 37% of the cohort, while HR+/HER2, HR+/HER2+, and HR-/HER2+ accounting for 22.2%, 14.8% and 7.4%, respectively. Five patients (18.5%) had no histopathological information. Of the 27 patients, the majority (77.8%) had a stage IV disease and 22.2% were at stage I-III. Ten patients (51.9%) were treatment-naïve and fourteen (52.9%) were previously treated. Twenty-four patients were sequenced with the OncoScreen panel (Burning Rock, Guangzhou, China) and 3 with a 108 breast-cancer related gene panel (PurePlasma, Burning Rock); 11 and 15 patients had tissue and plasma samples sequenced, respectively, and 1 patient had both sample types. TMB was only calculated for patients sequenced with the OncoScreen panel and showed a median value of 3.98 mutations/Mb. The median relative RTK fusion allele frequency (RTK. RAF) was 42.15% in this cohort.

**Table 1:** Clinicopathological and molecular characteristics of patients.

Characteristics	All patients (n = 27)
**Age, years**	
Median [IQR]	52.00 [40.00, 56.00]
**Molecular subtype, n (%)**	
HR+/HER2+	4 (14.8)
HR+/HER2-	6 (22.2)
HR-/HER2+	2 (7.4)
TNBC	10 (37.0)
NA	5 (18.5)
**Clinical stage, n (%)**	
I-III	6 (22.2)
IV	21 (77.8)
**Previous treatment, n (%)**	
No	10 (37.0)
Yes	14 (51.9)
NA	3 (11.1)
Chemotherapy	13 (48.1)
Endocrine therapy	4 (14.8)
HER2-targeted therapy	4 (14.8)
**Sample type, n (%)**	
Tissue	11 (40.7)
Plasma	15 (55.6)
Both tissue and plasma	1 (3.7)
**TMB, mutations/Mb**	
Median [IQR]	3.98 [2.74, 8.73]
RTK. RAF (fusion AF/max AF), %	
Median [IQR]	42.15 [15.75, 67.31]

TMB, tumor mutational burden; RTK. RAF, relative RTK fusion allele frequency; TNBC, triple-negative breast cancer; HR, hormone receptor; HER2, human epidermal growth factor receptor 2.

### Prevalence and Spectrum of RTK Fusions in Breast Cancer

**Figure 1:**
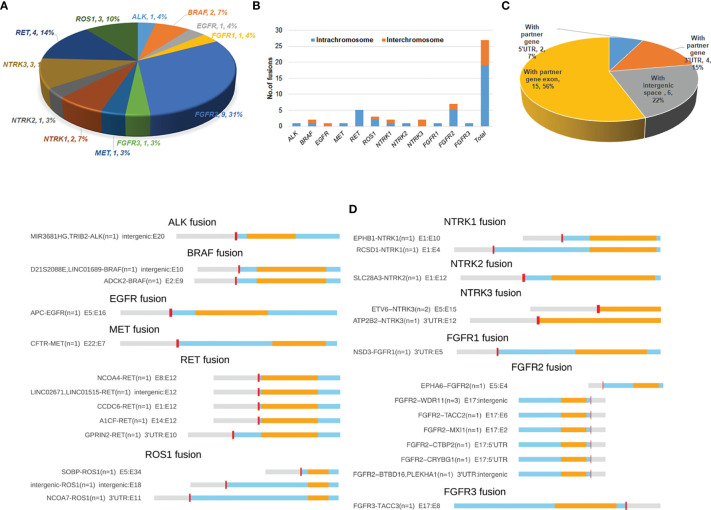
Distribution and spectrum of RTK fusions. **(A)** The distribution of RTK fusion events in different genes (n=29). **(B)** Distrubution of fusion variants by RTK genes and different chromosomal rearrangements (n=27); **(C)** RTK fusion variants classified by different types of fusion partner (n=27); **(D)** Spectrum of RTK fusions.

A total of 30 RTK fusion events were identified from 27 breast cancer patients with a prevalence of 1.875% (27/1440). Three patients harbored double fusions. Among the 30 evens, *FGFR2* fusions occurred most commonly (n=9), followed by *RET* (n=5), *ROS1* (n=3), *NTRK3* (n=3), *BRAF* (n=2), and *NTRK1* (n=2). Other RTK gene fusions including *ALK, EGFR, FGFR1, FGFR3, MET*, and *NTRK2* only occurred once (**Figure 1A**). The overall RTK fusion prevalence as well as fusion frequencies in different genes were comparable among different clinical stages and sample types (**Table S1**).

A total of 27 unique resultant fusions were discovered. The majority (n=19, 70.3%) of them were resulted from intrachromosomal translocation involving all 12 RTK genes except for *EGFR* and *NTRK3*, while interchromosomal fusions (n=8, 29.7%) only happened for *BRAF, ROS1, NTRK1, NTRK3* and *FGFR2* genes (**Figure 1B**). Twenty-one resultant fusions had the tyrosine kinase domain in-frame fused with a partner gene either at the 5’-end (n=16) or 3’-end (n=5), including 15 juxtaposed with an exon of the partner gene, 4 with the 3’-UTR (*GPRIN2-RET*, *NCOA7-ROS1, ATP2B2-NTRK3*, and *ESD3-FGFR1*), and 2 with the 5’-UTR of a partner gene (*FGFR2-CTBP2*, *FGFR2-CRYBG1*) (**Figures 1C, D**). We also observed 6 fusions of which the kinase domains were juxtaposed with an intergenic space (**Figures 1C, D**). Among the 27 fusions, *FGFR2-WDR11* (E17: intergenic) (n=3) and *ETV6-NTRK3* (E5:E15) (n=2) were recurrent (**Figure 1D**). The remaining fusions were only seen in one patient each. Of note, 1 patient harbored both *A1CF-RET* (E14:E12) and *GPRIN2-RET* (3’UTR: E10) fusions, and 2 out of the 3 patients identified with *FGFR2-WDR11* (E17: intergenic) harbored an additional *FGFR2* fusion: one with *FGFR2-BTBD16* (3’UTR: intergenic) and the other with *FGFR2-TACC2* (E17:E6). Of note, the vast majority of the 27 fusions we identified were rearranged with novel partners, with only 4 previously reported in breast cancer, including *ETV6-NTRK3*, *CCDC6-RET*, *NCOA4-RET* and *FGFR3-TACC3* (**Table 2**).

**Table 2:** List of RTK gene fusions previously reported in breast cancers.

RTK	Potential therapies	Fusion	Breast cancer subtype	Detected assay	Reference
NTRK3	Small molecule broad spectrum kinase inhibitors, NTRK inhibitors, IGF1R/INSR inhibitors	***ETV6-NTRK3***	Secretory BC	qRT-PCR, FISH, RNA-seq,	18, 19, 29;
ALK	ALK inhibitors	*EML4-ALK*	HER2+, luminal, and basal BC; TNBC inflammatory BC	RT-PCR, FISH	30, 31
RET	RET inhibitors	*ERC1-RET*	Breast invasive carcinoma;	RNA -seq	32, 33
		***CCDC6-RET***	ER- or HER2- BC	RNA-seq, DNA-seq	21
		***NCOA4-RET***	ER+/PR−/HER2+ breast cancer		
		*RASGEF1A-RET*	TNBC		
FGFR2	FGFR inhibitors	*FGFR2-AFF3*	Metastatic BC	RNA-seq, qRT-PCR	20
		*FGFR2-CASP7*	Metastatic BC; Breast invasive carcinoma	RNA-seq, qRT-PCR	20, 32, 33
		*FGFR2-CCDC6*	Metastatic BC; Breast invasive carcinoma	RNA-seq, qRT-PCR	20, 32
FGFR1	FGFR inhibitors	*ERLIN2-FGFR1*	Metastatic BC; Breast invasive carcinoma	RNA-seq, qRT-PCR	20, 32
		*WHSC1L1-FGFR1*	Not specified	RNA-seq	33
FGFR3	FGFR inhibitors	***FGFR3–TACC3***	TNBC	RNA-seq	34
BRAF	RAF kinase and MEK inhibitors	*KIAA1549-BRAF*	Breast carcinoma; Breast invasive ductal carcinoma	DNA-seq	35
		*BRAF–SND1*	HR+ BC	Anchored multiplex PCR, FISH	36
MET	MET inhibitors	*CAPZA2-MET*	Not specified	RNA-seq	33

Fusions in bold refer to those identified in the present study. RTK, receptor tyrosine kinase; BC, breast cancer; TNBC, triple-negative breast cancer; RT-PCR, reverse transcription-polymerase chain reaction; qRT-PCR, quantitative- reverse transcription-polymerase chain reaction; FISH, fluorescence in situ hybridization; HR, hormone receptor; HER2, human epidermal growth factor receptor 2; ER, estrogen receptor; PR, progesterone receptor.

**Figure 2:**
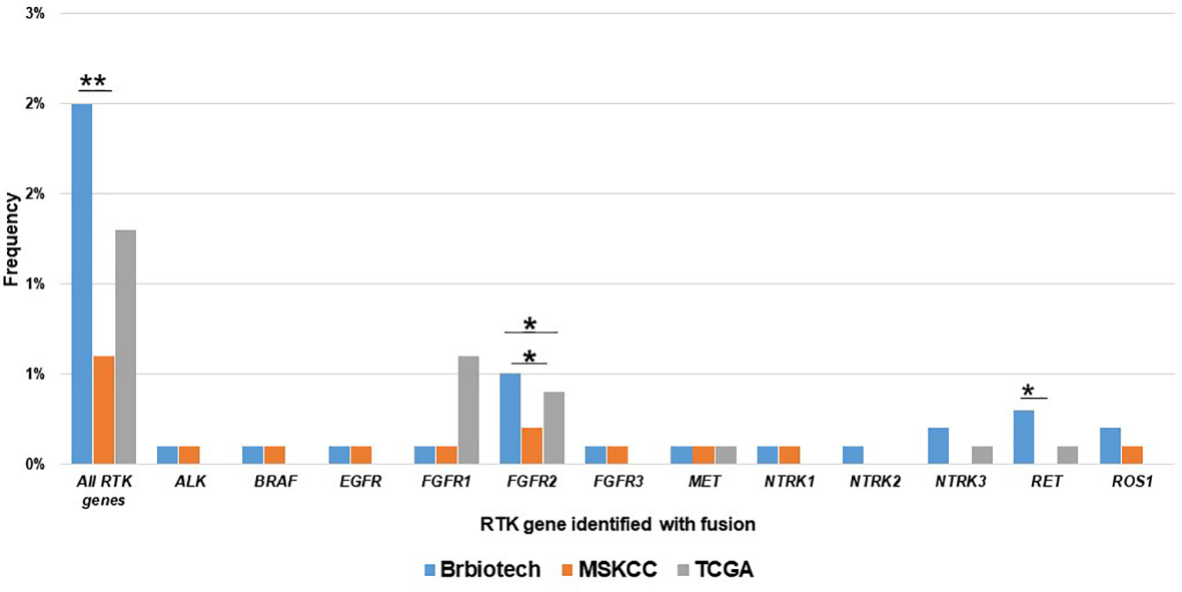
Comparison of the prevalence RTK fusions among different databases. Brbiotech (n=1440), MSKCC (n=1756), TCGA (n=996) *P-value <0.05; **P-value <0.01.

Next, we also compared the RTK fusion frequency among different databases. As shown in **Figure 2**, our cohort displayed higher overall RTK fusion (1.875% vs. 0.6%, P<0.001) and *RET* fusion (0.3% vs. 0%, P=0.021) frequencies than MSKCC (22). TCGA (0.6%) (23) revealed significantly more frequent *FGFR2* fusions than MSKCC (0.1%, P=0.017) as well as our cohort (0.1%, P=0.027). Other RTK genes did not show significant differences in fusion frequencies among databases.

### RTK Fusion Abundance Correlated With TMB

**Figure 3:**
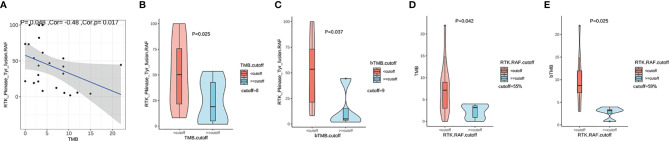
The correlation between TMB and relative RTK fusion allele frequency (RTK. RAF). **(A)** Pearson correlation between TMB and RTK. RAF (fusion AF/max AF) (n=24); **(B)** Comparison of RTK. RAF in TMB-high vs. TMB-low groups (n=24). **(C)** Comparison of RTK. RAF in blood TMB-high vs. blood TMB-low groups (n=13). **(D)** Comparison of the TMB in RTK. RAF -high vs. RTK. RAF -low groups (n=24). **(E)** Comparison of the blood TMB in RTK. RAF -high vs. RTK. RAF -low groups (n=13).

We evaluated the association between the RTK fusion abundance (defined as RTK. RAF) and TMB value and found a negative linear correlation (**Figure 3A**, r=-0.48, P=0.017). We defined the cut-off as the second tertile of TMB in the given cohort. Patients with TMB < 8 (Mutations/Mb) displayed a higher fusion abundance than those with TMB ≥ 8 (Mutations/Mb) (50.3%vs 19.0%, P=0.025, **Figure 3B**). Besides, in the eight TMB-high (>8 mutations/Mb) patients, four had received platinum-based chemotherapy; while only two out of the sixteen TMB-low (<8 mutations/Mb) patients had received platinum-based chemotherapy. In the subset of patients with plasma sample sequenced, using the cutoff of 9 (Mutations/Mb), patients with higher blood TMB (bTMB) also possessed lower fusion abundance than those with lower bTMB (5.0% vs 53.5%, P=0.037, **Figure 3C**). Similarly, patients with higher fusion abundance showed both significantly lower TMB (P=0.042, **Figure 3D**) and bTMB (P=0.025, **Figure 3E**). The phenomenon suggested a higher likelihood of subclonal nature for RTK fusions in TMB-high patients.

### Genomic Alterations Co-Occurring With RTK Fusions

**Figure 4:**
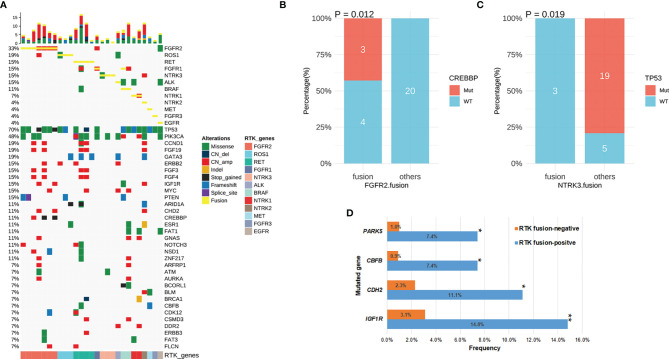
The concomitant mutations in RTK fusion-positive patients. **(A)** The oncoprint of 27 breast cancer patients with RTK fusion. **(B)** *CREBBP* mutation frequency in patients with *FGFR2* fusion vs. those with other RTK fusion. **(C)** *TP53* mutation frequency in patients with *NTRK3* fusion vs. those with other RTK fusion. **(D)** Comparison of mutation frequency in RTK fusion-positive vs. negative patients. *P-value <0.05; **P-value <0.01.

We next characterized the concomitant alterations in the 27 RTK fusion-positive breast cancers (**Figure 4A**). Fusions in different *RTK* genes were mutually exclusive except for one *BRAF* fusion-positive patient who also harbored a rearrangement of *FGFR1* fused with intergenic space. Of note, this fusion lacked the intact FGFR1 kinase domain therefore had been excluded from our analyses. Moreover, 4 out of the 7 *FGFR2* fusion-positive patients harbored *FGFR2* amplifications: three harbored *FGFR2-WDR11* (E17: intergenic) concomitant with another *FGFR2* fusion (*FGFR2-BTBD16* (3’UTR: intergenic), n=1; *FGFR2-TACC2* (E17:E6), n=1) or alone (n=1) and one had *FGFR2-CTBP2* (E17: 5’UTR). In addition, amplified *FGFR1* and *NTRK1* were also observed from the patient with *ESD3-FGFR1* (3’UTR: E5) and the one with *EPHB1-NTRK1* (E1:E10), respectively.

Concomitant genomic alterations in other genes were also comprehensively assessed in RTK fusion-positive cancers. *TP53* remained the most frequently mutated gene (70%) with the majority being missense mutations (12/19) (**Figure 4A**). *PICK3CA* alterations co-occurred the second most commonly (n=13, 48%). Other common concomitant alterations included amplifications in *CCND1*and *FGF19* (19%), as well as frameshift mutations in *GATA3* (19%).

In the RTK fusion-positive cohort, *CREBBP* mutation only co-occurred with *FGFR2* fusion (P=0.012, **Figure 4B**), while *NTRK3* fusion and *TP53* mutation were mutually exclusive (P=0.019, **Figure 4C**). By comparing the mutation frequency in RTK fusion-positive versus RTK fusion-negative breast cancer patients, we observed that *IGF1R* (14.8% vs. 3.1%, P=0.004), *CHD2* (11.1% vs. 2.3%, P=0.022), *CBFB* (7.4% vs. 0.9%, P=0.018) and *PAK5* (7.4% vs. 1.0%, P=0.024) mutated more commonly in the fusion-positive cohort (**Figure 4D**).

## Discussion

Our study comprehensively characterized the RTK gene fusions in Chinese breast cancer patients and identified 27 unique fusions that are potential oncogenic drivers. Among them, *ETV6-NTRK3*, *CCDC6-RET*, *NCOA4-RET* and *FGFR3-TACC3* have been reported in breast cancer previously*. ETV6-NTRK3* has been described as a primary oncogenic event in a rare subset of breast cancer secretory breast carcinoma (18, 19, 29). Clinical trials are currently ongoing that test the efficacy of entrectinib (a broad-spectrum kinase inhibitor for NTRKs, ROS, and ALK) in *NTRK*-rearranged solid tumors including breast cancer (NCT02568267, CT02097810). *CCDC6-RET* and *NCOA4-RET* have been previously characterized as oncogenic and occur recurrently in papillary thyroid and non-small cell lung cancers (37, 38). Recently, Paratala et al. identified *CCDC6-RET* (n=6) and *NCOA4-RET* (n=1) out of 9693 breast cancers. They also observed a rapid response to the RET inhibitor cabozantinib in a case with *NCOA4-RET*-positive breast cancer (21). Shaver et al. discovered *FGFR3-TACC3*, a canonical fusion across multiple solid tumors, in 1/80 TNBC tumors and *in vitro* studies indicate this fusion protein is a targetable driver in TNBC (34). *FGFR2-TACC2* that has been described in glioblastoma (39), NSCLC (40) and cervical cancer (41), was first identified in breast cancer in our study. **Table 2** also summarizes other previously reported RTK gene fusions in breast cancer that were not detected in our cohort. The expression of the canonical NSCLC *EML4-ALK* fusion was detected in 2.4% of breast cancers (30). Robertson et al. also identified *EML4-ALK* in 1/25 inflammatory breast cancers (31). Several studies profiling the landscape of kinase fusions across diverse cancers discovered *ERC1-RET*, *CAPZA2-MET*, and various *FGFR* fusions in breast cancer (20, 32, 33), of which *ERC1-RET*, *FGFR2-CASP7*, *FGFR2-CCDC6* and *ERLIN2-FGFR1* were recurrent. Besides, *KIAA1549-BRAF* was described in 2 breast cancers (35), and *BRAF-SND1* was identified in 2 hormone receptor-positive breast cancers (36).

Of note, we also discovered a variety of novel fusions including 16 with an unreported partner gene and 6 juxtaposed with an intergenic space (**Figure 1D**). Among them, *FGFR2-WDR11* (E17: intergenic), *FGFR2-BTBD16* (3’UTR: intergenic), *FGFR2-CTBP2* (E17: 5’UTR), *ESD3-FGFR1* (3’UTR: E5) and *EPHB1-NTRK1* (E1:E10) co-occurred with the amplification of the corresponding RTK gene. Although retaining the intact kinase domain, these amplicon-associated RTK fusions might represent the by-products of chromosomal amplifications known as passenger aberrations instead of oncogenic fusions (42). Therefore, their oncogenic significance merits further validation.

Intriguingly, we observed a negative correlation between relative RTK fusion abundance and TMB, suggesting that RTK fusions in TMB-low tumors are more likely to function as oncogenic drivers while fusion in TMB-high tumors are prone to be passenger alterations. Of note, in the eight TMB-high (>8 mutations/Mb) patients, four had received platinum-based chemotherapy; while only two out of the sixteen TMB-low (<8 mutations/Mb) patients had received platinum-based chemotherapy. The observation suggests the high mutation load is more likely to be caused by DNA damaging agent. Similarly in lung cancer, most driver mutations are found in non-smoking TMB-low NSCLC patients and high-TMB is associated with smoking history (43, 44). This can be explained by that the presence of an oncogenic driver is sufficient for the tumorigenesis in non-smokers while in patients with smoking history, tobacco carcinogens cause direct DNA damage and confer a high somatic mutation load that eventually increase the cancer risk (45, 46).

Our study has several limitations. Due to the retrospective nature of the study, we recuirted patients sequenced with un-uniform panels and diverse sample types. Enrolled patient were also with diverse clinical scenarios and a portion of them missed the clinical information. The heterogeneity may diminish the strength of the findings of our study. Our cohort was selected from patients who had underwent NGS, which tends to enroll more patients with advance disease, because patients with metastatic settings are more likely to seek for therapeutic option. Targeted DNA-based sequencing was used to detect RTK fusion in this study. Compared with RNA-based sequencing, this approach has certain technical limitations on detecting gene fusions. For instance, fusions with the breakpoint region insufficiently covered by the panel or those with breakpoint spanning repetitive sequence may not be identified (47). Therefore, this technique is likely to attenuate the capability of identifying unknown fusions and underestimate the prevalence of RTK fusions. Besides, DNA-based sequencing fails to provide direct evidence for the expression of resultant fusions at the mRNA level, so further evaluation of their transcripts is warranted to determine their significance. Moreover, the therapeutic information and clinical outcomes of patients were not provided, thus the therapeutic relevance of these potentially targetable RTK fusions remains unrevealed in breast cancer and merits further elucidation.

In conclusion, this is the first study comprehensively delineating the prevalence and spectrum of potentially targetable RTK fusions in Chinese breast cancers. Further study is ongoing to identify the enriched subpopulation who may benefit from RTK fusion inhibitors.

## Data Availability Statement

The original contributions presented in the study are included in the article/**Supplementary Material**. Further inquiries can be directed to the corresponding authors.

## Ethics Statement

The studies involving human participants were reviewed and approved by Fudan University Shanghai Cancer Center. Written informed consent for participation was not required for this study in accordance with the national legislation and the institutional requirements.

## Author Contributions

W-MC and XH contributed to the conception or design of the work. HX, KC, JZ, HZ, JS, JH, ZG, and HY contributed to the acquisition of data. ZT, JL, and TL contributed to the analysis and interpretation of data. ZT, JL, and TL drafted the MS. All authors contributed to the article and approved the submitted version.

## Funding

The authors are grateful for the financial grant from the National Science and Technology Major Project (2020ZX09201-013).

## Conflict of Interest

The authors declare that the research was conducted in the absence of any commercial or financial relationships that could be construed as a potential conflict of interest.

## Publisher’s Note

All claims expressed in this article are solely those of the authors and do not necessarily represent those of their affiliated organizations, or those of the publisher, the editors and the reviewers. Any product that may be evaluated in this article, or claim that may be made by its manufacturer, is not guaranteed or endorsed by the publisher.
