# Supplementary material for: Profiling Receptor Tyrosine Kinase Fusions in Chinese Breast Cancers
Source: Front Oncol. 2021 Sep 28;11:741142. doi: 10.3389/fonc.2021.741142 (PMC8506003; doi:10.3389/fonc.2021.741142)
Supplement: Supplementary file 2 [file Table_1.docx]

**Table S1.** The prevalence of RTK fusion among different stages and sample types.

|  | **Stage** | | | **Sample type** | | |
| --- | --- | --- | --- | --- | --- | --- |
| **Gene** | **I-III** | **IV** | **p-value** | **TIS** | **PLA** | **p-value** |
| **All RTK** | 2% (8) | 2.9% (19) | 0.506 | 1.2% (12) | 1.7% (16) | 0.424 |
| ***ALK*** | 0% (0) | 0.2% (1) | 1 | 0% (0) | 0.1% (1) | 0.966 |
| ***BRAF*** | 0.3% (1) | 0.2% (1) | 1 | 0.1% (1) | 0.1% (1) | 1 |
| ***EGFR*** | 0% (0) | 0.2% (1) | 1 | 0% (0) | 0.1% (1) | 0.966 |
| ***FGFR1*** | 0% (0) | 0.2% (1) | 1 | 0.1% (1) | 0% (0) | 1 |
| ***FGFR2*** | 0.8% (3) | 0.6% (4) | 1 | 0.3% (3) | 0.4% (4) | 0.91 |
| ***FGFR3*** | 0% (0) | 0.2% (1) | 1 | 0% (0) | 0.1% (1) | 0.966 |
| ***MET*** | 0% (0) | 0.2% (1) | 1 | 0% (0) | 0.1% (1) | 0.966 |
| ***NTRK1*** | 0% (0) | 0.3% (2) | 0.713 | 0.1% (1) | 0.1% (1) | 1 |
| ***NTRK2*** | 0.3% (1) | 0% (0) | 0.798 | 0.1% (1) | 0% (0) | 1 |
| ***NTRK3*** | 0.3% (1) | 0.3% (2) | 1 | 0.2% (2) | 0.1% (1) | 1 |
| ***RET*** | 0% (0) | 0.6% (4) | 0.299 | 0.1% (1) | 0.4% (4) | 0.321 |
| ***ROS1*** | 0.5% (2) | 0.2% (1) | 0.657 | 0.2% (2) | 0.1% (1) | 1 |
